# Supplementary material for: Detection, Identification, and Diffusion of Yeasts Responsible for Structural Defects in Provolone Valpadana PDO Cheese Using Multiple Research Techniques
Source: Foods. 2026 Jan 1;15(1):129. doi: 10.3390/foods15010129 (PMC12785867; doi:10.3390/foods15010129)

Figure S1. Provolone Valpadana cheese after 70 d of ripening, showing the diffused eye alteration along the cheese paste. The pH value of cheese interior is 5.38.

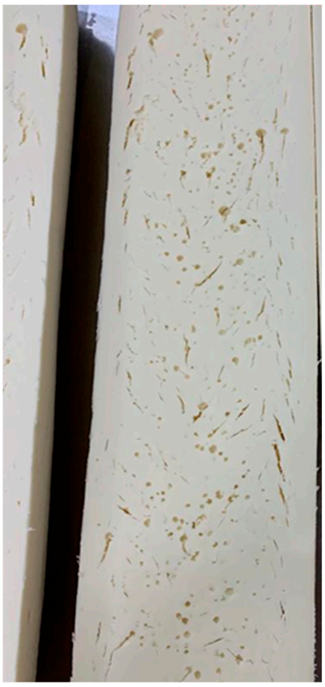

Figure S2. Typical shapes of Provolone Valpadana (PV), the salami (a) and the pear (b), mostly used for large distribution.

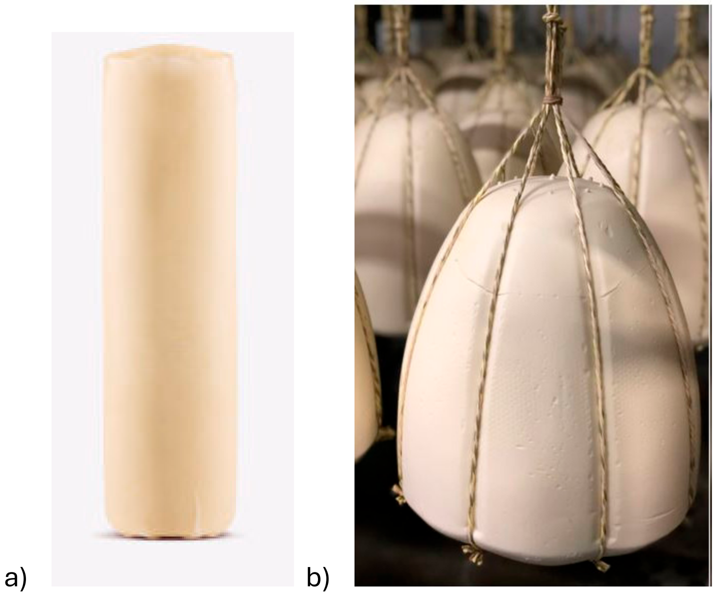

Supplement: Supplementary file 1 [file foods-15-00129-s001.zip › foods-4057389-supplementary.pdf]
